# EXamining the knowledge, Attitudes and experiences of Canadian seniors Towards influenza (the EXACT survey)

**DOI:** 10.1186/s12877-019-1180-5

**Published:** 2019-06-26

**Authors:** Melissa K. Andrew, Vladimir Gilca, Nancy Waite, Jennifer A. Pereira

**Affiliations:** 1 Division of Geriatric Medicine, Dalhousie University, 5955 Veterans’ Memorial Lane, Suite 3310 Veterans’ Memorial Building, Halifax, NS B3H 2E1 Canada; 2 Institut national de santé publique du Québec, Laval University, 2400 d’Estimauville, Quebec City, QC G1E 7G9 Canada; 3 School of Pharmacy, University of Waterloo, 10A Victoria St. S., Kitchener, ON N2G 1C5 Canada; 4 JRL Research & Consulting Inc., 1 Hurontario St., Suite 1605, Mississauga, ON L5G 0A3 Canada

**Keywords:** Influenza, Vaccine, Frailty, Older adults, Survey, Knowledge

## Abstract

**Background:**

Older adults are at high risk for influenza-related complications including worsening frailty and function. We surveyed older Canadians to explore the impact of influenza and determine how influenza knowledge influences vaccination decision-making.

**Methods:**

We disseminated an online survey through a national polling panel. The survey included questions about the respondents’ influenza vaccination practices and knowledge about influenza. Using validated measures, they reported their frailty and functional status prior to the 2016/17 influenza season, during illness (if applicable), and following the season. Regression analyses were used to examine predictors of poor functional outcomes.

**Results:**

Five thousand and fourteen adults aged 65 and older completed the survey; mean age was 71.3 ± 5.17 years, 42.6% had one or more chronic conditions, 7.8% were vulnerable and 1.8% were frail. 67.9% reported receiving last season’s influenza vaccine. Those who rarely/never receive the influenza vaccine were significantly less likely to correctly answer questions about influenza’s impact than those who receive the vaccine more consistently. Of the 1035 (21.5%) who reported experiencing influenza or influenza-like illness last season, 40% indicated a recovery longer than 2 weeks, and one-fifth had health and function declines during this time. Additionally, 3.1% of those afflicted “never fully recovered”. Older age, significant trouble with memory and having influenza/ILI were among the independent predictors of persistent declines in health and function.

**Conclusions:**

Given that frailty and function are important considerations for older adults’ well-being and independence, healthcare decision-makers must understand the potential for significant temporary and long-term impacts of influenza to make informed vaccine-related policies and recommendations.

## Background

Influenza is ranked among the 10 infectious diseases with the highest burden in Ontario [1]. Approximately half of influenza infections are asymptomatic or mild [2] and most who experience “influenza-like illness” (ILI; upper respiratory illness of sufficient severity to affect activities of daily living) will recover within 10 days [3]. However, others, particularly those 65 years of age and older, are at greater risk for a more detrimental impact through secondary medical complications, including pneumonia, cerebrovascular events, as well as exacerbation of underlying medical conditions [4]. Vulnerable older adults can also have persistent functional impairment following an episode of influenza; this “catastrophic disability” has lasting implications for the health and well-being of older adults and their families, and carries substantial resource implications for health systems [5]. As such, it is important to move beyond traditional consideration of influenza as a cause of short-term morbidity and mortality, to appreciate influenza’s health impact over longer time horizons.

In Canada, each province is responsible for developing its own immunization programs. Although the exact date of program initiation varies by year and by jurisdiction across the country, each province publicly funds the influenza vaccine for those 65 years and over, and most have done so for nearly two decades [6, 7]. The influenza vaccine is typically available in all provinces beginning in early-middle October, and residents can be vaccinated at any time before the end of the season but are encouraged to receive the vaccine as early as possible; the majority of individuals who opt to be vaccinated receive the vaccine by the end of December [8]. To protect the health of older adults, Canada’s National Advisory Committee on Immunization (NACI) has set a national target immunization rate of 80% [9] and yet vaccine uptake in seniors remains sub-optimal at 64–67% [10]. This is likely partially due to vaccine hesitancy created by reports that vaccine effectiveness (VE) is lower in older adults compared to younger populations [11–15]. However, this variability in VE is a source of debate [12] with recent research demonstrating that VE might also be significantly confounded by frailty, a measure of vulnerability to adverse health outcomes based on accumulation of health, mobility and functional deficits [16].

It is currently unknown whether Canada’s senior population is aware of the potential short- and long-term impacts of influenza, and if/how this affects their decision to receive the seasonal influenza vaccine. We therefore aimed to explore older Canadians’ experience of and views on influenza vaccination and illness through a survey of Canadian seniors. Understanding the impact of influenza on the health of older adults, as well as how the knowledge of this impact is linked to vaccination decision-making, could inform the development of more targeted tools and interventions to optimize vaccine uptake. Specifically, our objectives were to i) understand older adults’ self-reported experience with influenza, including increases in frailty and exacerbations of chronic conditions; and ii) evaluate knowledge, attitudes and behaviours towards influenza and influenza vaccines.

## Methods

In March/April 2017, we conducted an online quantitative survey (administered in either English or French) of Canadian seniors. The survey was called *EXamining the knowledge, Attitudes and experiences of Canadian seniors Towards influenza (EXACT).*

### Survey development

The survey was developed by the study team, consisting of influenza researchers and healthcare practitioners with expertise in public health, clinical medicine and pharmacy. The survey comprised multiple choice, true/false, and Likert scale of agreement questions. Respondents were asked to report their age, gender, province/territory of residence, community size, and chronic conditions.

Vaccination history was obtained by asking whether respondents received the influenza vaccine annually, whether they had received it this past season (2016/17), and at which location. As applicable, respondents were also asked why they opted for or decided against receiving the influenza vaccine.

Respondents were asked whether this past influenza season (defined as October 2016 to the time of survey completion), they had been told by a healthcare provider (HCP) that they had influenza, or whether they experienced an undiagnosed ILI that consisted of sore throat, fever, runny nose and cough. If they reported at least one experience, they were asked about recovery duration, and their use of health care services and medications (prescription and over-the-counter), and impact on chronic health conditions, frailty and function, for their most severe illness.

Frailty and function were measured using three validated scales, with permission. For frailty, we used the Canadian Study on Health and Aging’s Clinical Frailty Scale Version 1.2 (adapted for self-reporting) which features nine categories from “very fit” (score: 1) to “terminally ill” (score: 9) for a total range of one to nine [17]. For the purpose of this study, we defined a clinically meaningful change as any movement between categories. Function in basic activities of daily living (ADL) was measured by the Katz ADL Scale (includes bathing, dressing, toileting, transferring from bed or chair, bowel and bladder continence, and feeding oneself, with one point assigned for the ability to do each function independently) for a score range of zero to six [18]. Function in instrumental activities of daily living (IADL) was measured using the Older Americans’ Resources and Services (OARS) IADL (which includes telephone use, transportation, shopping, meal preparation, housework, managing medications, handling finances) and assigns points based on the ability to do these activities independently [2 points] or with help [1 point] for a score range of one to 14 [19–21]. Respondents were asked to report their frailty and function for three time points: before the 2016/17 influenza season (baseline), while they had influenza/ILI (for those who did), and current.

The survey also included questions on satisfaction with the currently available influenza vaccines, and knowledge and preferences of new influenza vaccines. Results were reported elsewhere [22].

The survey was tested for face validity and content validity in a sample of 10 Canadian adults 65 years and over.

### Recruitment

The survey was disseminated by Leger Marketing, a Canadian market research firm. The sample was generated from their Online Polling Panel of 400,000 Canadians initially recruited through a number of means: over the telephone (60%), referrals and affiliate programs (25%), partner programs (5%), offline recruitment (5%), social media (5%).

Participants were eligible if they were 65 years and older, and lived in Canada. Leger sampled proportionately to province population to achieve a sample size of approximately 5000 respondents. The sample size calculation was based on a question asking, “Which flu shot would you prefer to receive next year, assuming both vaccines are available for free?” We assumed that 25% of respondents would opt for standard-dose vaccine and 30% would select HD vaccine. To detect a 5% difference with 80% power, the total sample size required was 1356. We opted to recruit a larger sample to allow us to examine secondary outcomes. Sample size details have been previously reported [22].

### Statistical analysis

Data were analyzed overall, and by respondent demographics and vaccination status. We stratified respondents into two groups based on their annual vaccination behavior: those who always get the influenza vaccine/mostly get the influenza vaccine/get the influenza vaccine half the time vs. those who never get the influenza vaccine/rarely get the influenza vaccine. For each of the questions about influenza and influenza vaccine, we conducted chi-square tests to compare the proportion of respondents in each of the two groups who answered correctly. Two-sided tests were used for all statistical analyses. For each respondent, we used chi-square tests to compare the proportion of respondents who had upwards, downwards or no change in score for the three frailty and function scales from baseline to during influenza illness (if applicable) to post-influenza season. We initially attempted to model frailty and function scores using the raw scores in three separate ordinal logistic regression models. Based on the likelihood-ratio test of proportionality of odds (*p* > 0.0001), the assumptions of the models were invalid. Therefore, the scores were dichotomized into worsened score (coded as 1) vs. same or improved score (comparing baseline to post-influenza season scores; coded as 0) as the outcomes for a logistic regression model for each of the three scales in use. Logistic regression was performed to determine whether having influenza or ILI (predictor variable) was associated with worsened frailty or function (outcome variable), after adjusting/controlling for variables that could be associated with increased risk of influenza, its complications, or general health declines: increased age, sex, comorbidities (asthma, COPD, blood disorder, heart disease, kidney disease, liver disease, neurological disease, cancer and HIV [23–25]. All comorbidities were coded as absent (0) or present (1), while sex was coded as male (0) or female (1). Age was coded as a continuous variable. Variables that were collinear were removed from the model. Analyses were done using STATA 10.0 (2007, StataCorp, LP, College Station, TX).

This study was approved by the Nova Scotia Health Authority’s Research Ethics Board.

## Results

Between March 20th and April 5th, 2017, completed surveys were collected from 5014 people 65 years and older, with representation from all ten Canadian provinces (Table 1). The mean age of the respondents was 71.3 ± 5.17 years (range = 65–96), 50% were male, 42.6% had one or more chronic conditions, and 9.6% considered themselves vulnerable or frail. The median time to complete the survey was 14 min. Overall response rate was 30.4%.

**Table 1:** Baseline characteristics of respondents (*n* = 5014)

Characteristic	n (%)
Age	
Mean age (yrs); SD	71.34; 5.17
Median age	70
65–74 years	3803 (75.8)
≥ 75 years	1211 (24.2)
Gender	
Man	2507 (50.0)
Woman	2505 (50.0)
Other (i.e. transgender)	2
Province	
British Columbia	711 (14.2)
Alberta	435 (8.7)
Saskatchewan	144 (2.9)
Manitoba	166 (3.3)
Ontario	1914 (38.2)
Quebec	1258 (25.1)
New Brunswick	124 (2.5)
Nova Scotia	154 (3.1)
Prince Edward Island	23 (0.5)
Newfoundland and Labrador	85 (1.7)
Location	
Village (< 1000 people)	417 (8.3)
Town (1000 to 99,999 people)	1749 (34.9)
City (> 100,000 people)	2848 (56.8)
Language	
English	3600 (71.8)
French	1414 (28.2)
Chronic condition	
None	2877 (57.4)
Diabetes	906 (18.1)
Heart disease	551 (11.0)
Asthma or chronic lung disease other than COPD	411 (8.2)
Blood disorders (not including high or low blood pressure)	326 (6.5)
COPD	260 (5.2)
Cancer	220 (4.4)
Neurological disorders	130 (2.6)
Kidney disease	122 (2.4)
Significant trouble with memory	83 (1.7)
Liver disease	36 (0.7)
HIV/AIDS	6 (0.1)
Grouped Frailty Level	
Very fit/well	3076 (61.3)
Managing well	1461 (29.1)
Vulnerable	389 (7.8)
Mildly-severely frail or terminally ill	88 (1.8)
Grouped Katz ADL level	
Some dependence	134 (2.7)
Completely independent	4880 (97.3)
Grouped OARS IADL level	
Some dependence	592 (11.8)
Completely independent	4422 (88.2)

This table summarizes respondents’ demographics, medical history and frailty and function levels at the time of survey completion

### Vaccination behaviour

The majority (3207, 64%) of respondents receive the influenza vaccine annually, 858 (17.1%) do not follow a regular practice, and 949 (18.9%) never receive it.

**Fig. 1:**
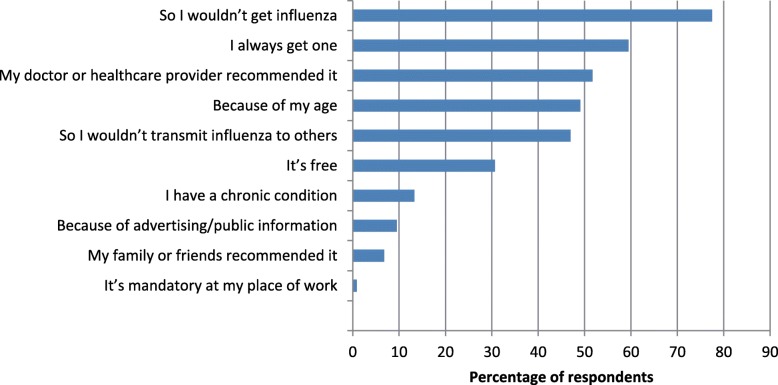
Reasons for receiving the influenza vaccine in the 2016/2017 season. Respondents who received the influenza vaccine during the 2016/17 season (*n* = 3403) were asked why they opted for it, and were provided with a list of possible reasons as well as the ability to provide one or more reasons not listed

This past season, 3403 (67.9%) of respondents were vaccinated against influenza. The primary reasons for receiving the vaccine were: to prevent influenza, out of routine, and because their HCP recommended it (Fig. 1).

Respondents who were *vulnerable* were significantly more likely to be vaccinated (76.5%) than those in the two healthier Frailty Scale categories of *very fit/well* (66.0%, *p* < 0.001) and *managing well* (69.3%, *p* = 0.007).

The majority of respondents received the influenza vaccine at their family physician’s office or at a pharmacy (36.8 and 34.2%, respectively), while 11.4% received the vaccine at a public health clinic. The remainder of patients (17.6%) were vaccinated at community health centres, walk-in clinics, senior homes, and senior centres.

The most popular reasons for abstaining from the influenza vaccine was thinking that it was unnecessary (39.2%), thinking it was ineffective (26.9%), concern over side-effects (22.9%), and disliking injections (13.3%). Less common rationales included forgetting to receive the vaccine (7.7%), clinic logistic issues (2.9%), recommendations from HCPs or family/friends against receiving it (2.2 and 3.0%, respectively) and contraindications to receiving the vaccine (1.1%).

### Impact of influenza/ILI

Of all respondents, 245 (4.9%) indicated that they had been diagnosed with influenza and 790 (16.6%) indicated that they had experienced ILI. Among these 1035 individuals, 39.3% reported that their recovery took longer than 2 weeks, and 3.1% reported “I never fully recovered”. During their illness, 480 (46.4%) sought over-the-counter or prescription treatment from a pharmacy, 324 (31.3%) respondents visited their family doctor, 207 (20.0%) visited a walk-in clinic, 15.7% went to the ER and 13.9% were admitted to hospital.

**Fig. 2:**
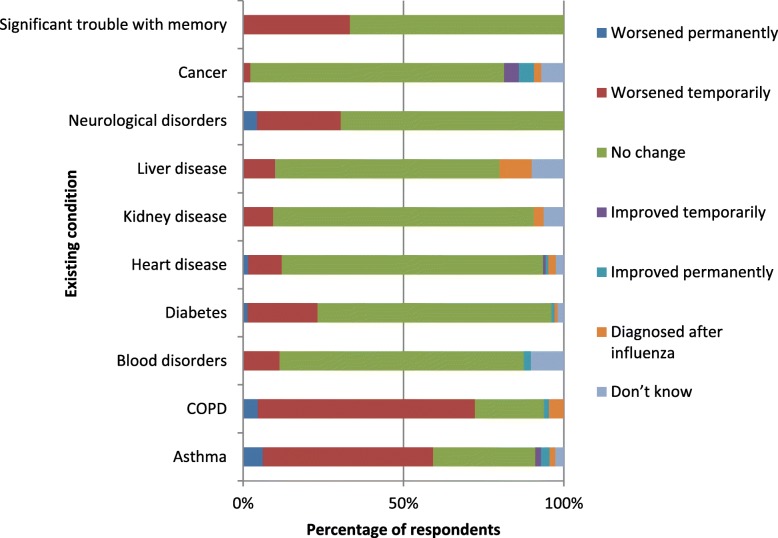
Respondents’ report of the impact of influenza on existing conditions. For each of their existing conditions, respondents who reported having influenza or ILI during the 2016/2017 season indicated how the condition was affected, if at all, during their period of illness

A significant proportion of respondents had their pre-existing conditions worsen while they had influenza/ILI (Fig. 2). This was particularly true of lung conditions: 53.1% of those with asthma and 67.7% with COPD had exacerbated symptoms while ill; 6.2 and 4.6% of individuals with these respective conditions had a decline lasting until the time of survey completion.

Having influenza/ILI was associated with a decline in ability to perform ADL at time of illness, though overall respondents appeared to return to baseline levels after recovery. Of those who had influenza/ILI, 118 (11.5%) relied on others for help with IADL prior to the influenza season, and this number increased to 314 (30.3%) during illness. The proportion of respondents who required help for some basic ADL, increased from 3.3% prior to the influenza season to 5.5% during influenza/ILI.

Increased age, having influenza/ILI, and having significant memory trouble prior to influenza were all significantly and independently associated with higher odds of worsened frailty, IADL function and function in basic ADLs post-influenza season (Table 2). Liver disease, neurological disease and heart disease were independently associated with declines in two of the three scales, while asthma and kidney disease were associated with a decline in a single scale only (OARS ADL).

**Table 2:** Association of patient factors and medical conditions with worsening frailty and function

	Frailty Scale^b^		OARS IADL Scale^b^		KATZ ADL^b,c^	
	OR (95% CI)	*P*-value	OR (95% CI)	*P*-value	OR (95% CI)	*P*-value
Age	1.05 (1.02, 1.07)	<0.001*	1.06 (1.03, 1.09)	< 0.001*	1.08 (1.01, 1.17)	0.03*
Sex^a^	1.10 (0.95, 1.26)	0.20	1.07 (0.91, 1.27)	0.39	1.17 (0.74, 1.87)	0.50
Influenza/ILI	2.70 (2.03, 3.60)	<0.001*	1.89 (1.33, 2.68)	<0.001*	2.68 (1.07, 6.73)	0.04*
Asthma	1.33 (0.85, 2.07)	0.21	1.89 (1.19, 2.99)	0.01*	0.54 (0.07, 4.13)	0.55
COPD	1.52 (0.91, 2.55)	0.11	1.57 (0.88, 2.77)	0.12	0.80 (0.10, 6.22)	0.83
Blood disorder	1.20 (0.73, 1.96)	0.47	1.21 (0.70, 2.08)	0.50	2.32 (0.71, 7.55)	0.16
Heart disease	1.18 (0.78, 1.78)	0.44	1.77 (1.16, 2.68)	0.01*	2.93 (1.04, 8.29)	0.04*
Kidney disease	1.12 (0.52, 2.40)	0.77	2.07 (1.06, 4.03)	0.03*	N/A	N/A
Liver disease	3.38 (1.26, 9.08)	0.02*	3.27 (1.09, 9.79)	0.03*	N/A	N/A
Neurological disease	1.95 (1.00, 3.83)	0.05*	3.58 (1.96, 6.56)	<0.001*	2.14 (0.27, 16.75)	0.47
Cancer	1.53 (0.84, 2.77)	0.16	1.27 (0.62, 2.58)	0.51	2.39 (0.52, 10.94)	0.26
Significant trouble with memory	2.33 (1.08, 5.02)	0.03*	4.28 (2.13, 8.58)	<0.001*	10.43 (2.82, 38.63)	<0.001*

This table shows the associations of patient demographics and comorbidities with declines in frailty and function at the end of the influenza season

*p ≤ 0.05, logistic regression models

^a^Data from the respondents who identified themselves as transgender were not included in the model due to the low sample size (*n* = 2)

^b^HIV was dropped from the model due to collinearity

^c^Kidney disease and liver disease were dropped from the model due to collinearity

### Knowledge about influenza

Respondents indicated a mixed level of knowledge with respect to influenza (Table 3). While 90.8% understood that seniors are at a higher risk for influenza complications than younger adults, only 42.9% were aware of influenza’s impact on heart attack risk, 39.2% believed that you can get influenza from the vaccine, and more than half (50.9%) thought that the vaccine is more effective in seniors than young adults.

**Table 3:** Number and percentage of respondents who answered influenza-related True/False questions correctly

Statement	All respondents (*n* = 5014)	Gets influenza vaccine always/ mostly/half the time (*n* = 3666)	Rarely or never gets influenza vaccine (*n* = 1348)	*P*-value*
There is no difference between having a cold and having influenza	4493 (89.6%)	3309 (90.3%)	1184 (87.8%)	< 0.01
People who are 65 years and older are at higher risk for influenza complications	4555 (90.8%)	3455 (94.2%)	1100 (81.6%)	< 0.001
If you already have heart or lung problems, influenza can make them worse	4218 (84.1%)	3107 84.8%	1111 82.5%	< 0.05
If you already have heart or lung problems, influenza can increase your risk of death	4023 (80.2%)	2983 81.4%	1040 77.2%	< 0.001
Influenza can put those 65 years and older at a greater risk for a heart attack	2151 (42.9%)	1647 44.9%	504 37.4%	< 0.001
Those 65 years and older who are hospitalized with influenza are at a higher risk of developing further complications than those not hospitalized	2728 (54.4%)	1987 54.2%	741 55.0%	0.286
Those 65 years and older with influenza always fully recover	3390 (67.6%)	2579 70.3%	811 60.2%	< 0.001
You can get influenza from the influenza vaccine	3048 (60.8%)	2591 70.7%	457 33.9%	< 0.001
The influenza vaccine is more effective in those 65 years and older than in younger adults.	2460 (49.1%)	1895 51.7%	565 41.8%	< 0.001

This table compares respondents’ knowledge about influenza and influenza vaccines based on whether the respondent receives the influenza vaccine regularly (every year, most years or half of the time), or not (rarely or never receives the vaccine). *chi-square tests

Respondents who never or rarely receive the influenza vaccine were significantly more likely to answer incorrectly to eight of the nine questions than those who annually receive the influenza vaccine at least half of the time. The largest difference was observed in a question regarding influenza vaccine: 33.9% of those who never/rarely receive the vaccine vs. 70.7% of all other respondents correctly indicated that it is not possible to get influenza from the vaccine.

## Discussion

In our survey of 5014 Canadian seniors, two-thirds reported having been vaccinated with last season’s influenza vaccine although many were unaware that they are at risk for influenza-related complications, and held misperceptions about influenza vaccines. One in five respondents reported that they had influenza or ILI in the 2016/17 season; among those individuals, more than 40% took over 2 weeks to recover and 13.9% were hospitalized. While our sample was relatively fit and healthy at baseline, individuals who had influenza/ILI reported declines in function and worsening frailty during their acute illness phase, and for 3.1%, this persisted after “recovery”. Older age, significant trouble with memory, and having influenza/ILI were among the independent predictors of declines in health and function.

We recruited a large sample, with respondents from across Canada. The immunization rate observed in this study (67.9%) is similar to a recently reported rate of 64% [26], lending support to our sample’s representativeness. We observed that having had influenza/ILI this past season was significantly associated with lasting frailty and functional impairment, and that respondents reported high healthcare utilization, seeking influenza care and treatment at pharmacies, family physicians’ offices, ER departments, and even as in-patients. Most reported that worsening function and frailty were confined to the period of influenza illness/ILI. However, this temporary loss of independence is not inconsequential; the inability to do routine daily tasks translates to an increased need for assistance, which is burdensome for older adults who live alone or are themselves caregivers. Future work is required to further investigate the associations that we have identified, in order to establish whether it is truly a causal relationship between the influenza illness and the impaired frailty and function, or whether these could be attributable to confounding factors that lead those afflicted to more often experience these declines. Additionally, examining the long-term impact of influenza in a frailer group of seniors would help determine whether our observations are simply “the tip of the iceberg”. The literature suggests that a substantial subset of frail older adults emerge from an acute illness such as influenza with persistently worse function than prior to illness [5, 27]. We also found that significant memory troubles and several chronic conditions were important independent predictors of persistently worse frailty and function following the influenza season, highlighting the vulnerability of older people with these conditions to health declines and the need for interventions targeting those most at risk for influenza-related complications.

Most respondents indicated that they opted for the influenza vaccine because their HCP recommended it, which supports previous findings [28]. Similar to past studies, we observed that the most common reasons for not receiving the influenza vaccine were not believing that influenza was severe or that the vaccine was effective [28–30]. Our study demonstrates that seniors hold misconceptions regarding influenza and influenza vaccine, particularly those who do not typically opt for annual influenza vaccination. This corroborates previous studies finding that adults who recognized influenza’s severity and perceived themselves as “high-risk” were more likely to accept the vaccine [31]. This may also be linked to psychological factors – a German study showed that feeling high levels of stress is positively associated with opting for influenza vaccination [32]; it is possible that believing oneself to be at greater risk for influenza-related complications leads to significant stress. There are evident knowledge gaps preventing individuals from making optimal vaccine-related decisions. These gaps will gain in importance over time –our survey sample is an aging cohort who may become tomorrow’s frail older adults. Future work is needed to increase education, promote awareness and combat myths of the clinical consequences of influenza in the senior population and the risk/benefit profile of influenza vaccines.

Our study had limitations. Our response rate of 30.4% is fairly standard for online surveys, but it is possible that those who responded may have different health experiences from those who did not. To understand the impact of this potential bias, we have compared our findings to the literature, where possible, and have noted strong similarities, lending credibility to our results. Our sampling frame is unlikely to have included very frail Canadians, or those who suffered long-term consequences of influenza resulting in hospitalization or residing in long-term care facilities (LTCF) at the time of survey completion. However, given that only 4% of Canadians reside in LTCF [33], we expect that this exclusion had minimal effect on our estimation of the overall impact of influenza in seniors. Further studies are needed to focus on these populations. Our online panel sample may have over-represented cognitively-well older adults. Our survey may also have appealed to people with strong views on vaccination or desire to share illness experiences. However, we did have responses from all Canadian provinces, with a wide age range (24.2% aged 75+) and gender parity. No data on the survey non-responders were collected, and therefore it is not possible to state whether we were able to completely eliminate potential non-response bias. However, we implemented several measures including pilot-testing the survey with 10 older adults and having the survey open for 3 weeks, to ensure that the survey was easy to understand and to facilitate participation. Since the survey took place just after the season, there is potential for recall bias. Additionally, respondents may have had ILI caused by other respiratory viruses, overestimating the incidence of influenza and also affect the data around clinical impact. To limit this effect, we asked whether the respondent had a clinical diagnosis, and provided a restrictive set of criteria for ILI. Our reported influenza rate of 4.9% is similar to laboratory-confirmed influenza rates previously observed in senior populations [34, 35], while our ILI rate of 16.6% corroborates previous findings [36]. Finally, our survey is subject to the same type of biases as others using a self-report design. We aimed to minimize the probability of a recall bias with the relatively short period between the events and their assessment, and the wording of the questions which was designed to cue recall of reportable events. We were unable to confirm patient responses through chart audits, so recall bias and issues with cognitive impairment remain a possibility. However, previous studies which have compared the reports of seniors to their medical charts have found seniors to have reliable memories, particularly for events which required a visit to a healthcare provider [37].

## Conclusion

Our results show that while a majority of older adults receive the yearly seasonal influenza vaccine, coverage rates and knowledge about influenza illness and vaccine remain suboptimal. Those with influenza/ILI reported functional declines and worsening frailty during their illness, and some have not fully recovered. Further research is required to assess this association. It is important to ensure that Canadian seniors (along with clinicians and policy-makers) have accurate information to make vaccine-related recommendations which would facilitate increased vaccine uptake and consequently optimize the health protection of this high-risk population. This includes the understanding that recovery from influenza can be prolonged, incomplete, and increase the burden on patients and caregivers. Given that frailty and function are closely linked to older adults’ wellbeing and independence, it is critical to consider influenza’s impact on these health outcomes when designing future research studies and public health programs for influenza prevention.

## Data Availability

The datasets generated and analysed during the current study are not publicly available given that study participants did not consent for their individual data to be shared (aggregate data sharing only), and the possibility that a subset of participants may be identified from their responses which would compromise anonymity.

## Abbreviations

EXACT: EXamining the Knowledge, Attitudes and Experiences of Canadian Seniors Towards Influenza
HCP: Healthcare personnel
IADL: Instrumental activities of daily living
ILI: Influenza-like illness
LTCF: Long-term care facility
NACI: National Advisory Committee on Immunization
OARS: Older Americans’ Resources and Services
VE: Vaccine effectiveness
